# Validation of PCR-based protocols for the detection of *Echinococcus multilocularis* DNA in the final host using the Intestinal Scraping Technique as a reference

**DOI:** 10.1016/j.fawpar.2019.e00044

**Published:** 2019-03-10

**Authors:** P. Maksimov, M. Isaksson, G. Schares, T. Romig, F.J. Conraths

**Affiliations:** a Friedrich-Loeffler-Institut, Federal Research Institute for Animal Health, Institute of Epidemiology, Südufer 10, 17493 Greifswald-Insel Riems, Germany; b Department of Virology Immunobiology and Parasitology, National Veterinary Institute, Uppsala, Sweden; c FG Parasitologie 220B, Universität Hohenheim, 70593 Stuttgart, Germany

**Keywords:** *Echinococcus multilocularis*, Diagnosis, Magnetic capture, PCR, Intestinal Scraping Technique, Faecal PCR inhibitors

## Abstract

Oral uptake of infectious *Echinococcus multilocularis* eggs shed by canids with their faeces may lead to development of alveolar echinococcosis in humans, which is clinically similar to a malignant infiltrative tumor and may be fatal if left untreated. *E. multilocularis* is therefore regarded as one of the most important and neglected metazoan parasites in the Northern hemisphere. The diagnosis of this tapeworm in the final host plays a key role in the epidemiology of *E. multilocularis*.

The diagnostic performance of a magnetic-capture (MC) DNA extraction protocol in combination with a minor groove-binder real time PCR (MC-MGBqPCR) for the detection of *E. multilocularis* eggs was determined relative to a highly sensitive variant of the Intestinal Scraping Technique (IST) using faecal samples of foxes. In addition, we compared results obtained by MC-MGBqPCR with those of a previously validated protocol (QIAamp Fast DNA Stool Mini Kit (QT) combined with a TaqMan qPCR). Furthermore, a workflow using the NucleoMagVet DNA extraction kit (NM) in combination with MGBqPCR and TaqMan-qPCR was also included in the comparisons.

To estimate the analytical sensitivity, phosphate-buffered saline and fox faecal samples were spiked with different numbers of eggs and tested in defined combinations of DNA extraction and PCR protocols. To assess the diagnostic sensitivity of the different workflows, samples were used that had been collected from the ampulla recti or the rectum of 120 foxes hunted in Brandenburg, Germany. The samples represented five IST categories formed according to the *E. multilocularis* worm burden of the foxes. For DNA extraction by MC or using two other commercial extraction kits, the supernatants obtained from 3 g of bead-beaten faecal samples were used. The extracted DNAs were then processed in the respective PCR protocols.

The MC-MGBqPCR showed the highest diagnostic sensitivity (93%; 95% Confidence Interval (CI): 86–97%) relative to IST. The QT extraction protocol in combination with TaqMan-qPCR had the second highest sensitivity (89%; 95% CI: 80–94%), followed by NM with MGBqPCR (86%; 95% CI: 77–93%) in comparison to IST. The lowest diagnostic sensitivity was found for the NM combined with the TaqMan-qPCR protocol (72%; 95% CI: 62–82%).

In conclusion, the MC-MGBqPCR seems to represent a suitable alternative to IST. However, applied to 3 g faecal samples, the less costly QT-TaqMan-qPCR workflow yielded a similar diagnostic sensitivity relative to IST. However, differences between these two workflows were not statistically significant.

## Highlights

• PCR based protocols can be used for the detection of *E. multilocularis* in faeces of final hosts after careful validation
• A DNA capture method seems to represent a suitable alternative to the Intestinal Scraping Technique
• The QT-TaqMan-qPCR workflow yielded also a similar diagnostic sensitivity relative to the Intestinal Scraping Technique
• None of the DNA extraction kits was able to remove faecal PCR inhibitors completely

## 1 Introduction

*Echinococcus multilocularis* is regarded as one of the most dangerous zoonotic metazoan parasites in the northern hemisphere (Eckert et al., 2000). The parasite uses canids as definitive hosts, i.e. the development of adult worms takes place in the intestine of these animals resulting in the shedding of mature worm eggs either packaged in the gravid proglotids or dispersed within faeces (Kapel et al., 2006). Cats can also be regarded as potential definitive hosts of *E. multilocularis*, but there are indications that they play a minor role in the lifecycle of the parasite (Thompson et al., 2003; Kapel et al., 2006). Worm eggs represent the infectious stage for intermediate hosts, which include a wide spectrum of species, among which rodents, e.g. Arvicolidae, play a prominent role (Woolsey et al., 2015a; Woolsey et al., 2015b). Successful infection of an intermediate host leads to the development of larval stages (metacestodes) of *E. multilocularis* in different organs, but mainly in the liver, causing alveolar echinococcosis (AE) (Matsumoto et al., 2010). The development of AE is similar to that of a malignant tumor in the sense that the metacestode grows in an ‘infiltrative’ fashion and may ‘metastasize’, if metacestode tissue is transported to other organs via the lymph or blood stream (Matsumoto et al., 2010). Humans accidentally infected with *E. multilocularis* by oral uptake of infectious eggs serve as an aberrant (dead-end) intermediate hosts. While AE is still a rare disease in humans in Europe (Gottstein et al., 2015), an increasing prevalence has been reported (Schweiger et al., 2007).

Several diagnostic methods are available to monitor *E. multilocularis* infections in the final hosts or to verify freedom from the parasite in a population. Coproantigen ELISAs were applied in several studies to estimate the prevalence of *E. multilocularis* infections in the final host (Conraths and Deplazes, 2015). The method allows to screen large numbers of faecal samples, but the positive predictive value of this test may be reduced in populations with a low *E. multilocularis* prevalence (Torgerson and Deplazes, 2009). Moreover, the specificity of the test seems to vary (Conraths and Deplazes, 2015). Flotation-based protocols are broadly applied for the detection of *E. multilocularis* eggs. However, this classical method has several diagnostic disadvantages. Since tapeworm eggs of the family of Taeniidae cannot be morphologically distinguished, it is not possible to detect *E. multilocularis* eggs directly using this method. It is therefore necessary to differentiate taeniid eggs on the DNA level for species determination. Furthermore the diagnostic sensitivity of the flotation-based protocols in detecting taeniid eggs is only about 50% (Liccioli et al., 2012).

The Sedimentation and Counting Technique (SCT) and Intestinal Scraping Technique (IST) are widely used methods and regarded as reference standards for the diagnosis of *E. multilocularis* in its main definitive host, the red fox (*Vulpes vulpes*) (Eckert et al., 2001; Tackmann et al., 2006; Conraths and Deplazes, 2015). The advantages of these methods are the possibility to analyze the worm burden in the intestines quantitatively and a very high specificity of almost 100% (Tackmann et al., 2006; Umhang et al., 2011). The diagnostic sensitivity of SCT and IST has been estimated at around 83%, but this value may depend on several factors such as the condition of the intestines, i.e. potential autolysis, long freezing periods, details of the protocol and the experience of the laboratory personal. The main disadvantage of these methods is that they can only be applied to samples collected during the necropsy of definitive hosts, i.e. post mortem. They are therefore hardly suitable to monitor populations of pet dogs and cats for *E. multilocularis* infections. SCT and IST are also laborious and can only be performed under special safety requirements by experienced staff (Tackmann et al., 2006; Umhang et al., 2011; Conraths and Deplazes, 2015).

Detection of *E. multilocularis* copro-DNA, extracted from proglotids, eggs or parts of parasitic tissue dispersed in the faecal samples may represent an interesting alternative to SCT and IST (Conraths and Deplazes, 2015; Maksimov et al., 2017). The big advantage of DNA-based protocols is the high throughput of samples, potential intra vitam diagnosis of patent *E. multilocularis* infections in the final host and the chance to use the amplified DNA fragments for a genetic characterization of the detected parasite isolate. However, the sensitivity of these methods depends strongly on the DNA extraction protocol and on its ability to remove PCR inhibitors, which are often present in faecal samples (Maksimov et al., 2017). Furthermore, only small amounts of faecal matter (up to 500 mg) can be processed if the target DNA is extracted using commercial kits. Heterogeneous distribution of proglotids or parasite eggs in a faecal sample can also lead to false-negative test results (Maksimov et al., 2017). Hence, processing a larger amount of faecal matter for DNA extraction may increase the probability of detecting *E. multilocularis* DNA in faecal samples. However, DNA extraction from a larger amount of faecal matter may lead to an enrichment of copro-inhibitors, thus resulting in false-negative PCR results. To overcome this problem, targeted capture of parasite DNA was applied in recent studies (Isaksson et al., 2014; Maas et al., 2016; Wahlstrom et al., 2016).

With the available and published protocols, up to 3 g of faecal sample can be used for the extraction of target DNA, thus increasing the detection probability of *E. multilocularis* DNA in faecal samples (Isaksson et al., 2014; Oines et al., 2014; Maas et al., 2016). A magnetic capture (MC) probe-based DNA extraction method combined with a minor groove binder (MGB) hydrolysis probe real time PCR (MGBqPCR) was validated using faecal samples from foxes that had been tested by SCT as a reference (Isaksson et al., 2014). Since IST is a widely used protocol to diagnose *E. multilocularis* infections in foxes, we validated the MC-MGBqPCR workflow against a sensitive and specific IST protocol (Tackmann et al., 2006). In addition, we included two commercially available DNA extraction kits and the respective real time PCR protocol in the analysis.

## 2 Material and methods

### 2.1 Preparation of samples spiked with defined numbers of eggs

To assess the performance of the protocols without the influence of potentially inhibitory substances that must be expected in faecal samples, a defined number of eggs was added to phosphate-buffered saline (PBS). To investigate the effect of faecal matter on the test protocols, we used faecal samples spiked with defined numbers of *E. multilocularis* eggs isolated from the faeces of naturally infected foxes hunted in the federal state of Brandenburg, Germany in 2008. We used samples spiked with one (n = 10), two (n = 10), four (n = 10), eight (n = 10), 16 (n = 5) and 32 (n = 5) *E. multilocularis* eggs. These samples were prepared as described by Oines et al. (2014).

Briefly, a stock suspension of *E. multilocularis* eggs in PBS was pipetted into a Petri dish and defined numbers of eggs aspirated using a 10 μl pipette under a microscope (Nikon GmbH, Düsseldorf, Germany). The drop with the aspirated eggs was pipetted onto a piece of plastic foil cut from waste disposal bags (Sarstedt Nümbrecht, Germany) (appr. 0.5 × 0.5 cm) in an empty Petri dish. The number of eggs present on the plastic foil was checked under the microscope. The piece of plastic foil with the sample was then transferred into a 15 ml Falcon tube containing 0.8 g of 0.5 mm zirconium oxide beads (BioSpec, Bartlesville, USA), 0.6 g of 2 mm zirconium beads (BioSpec, Bartlesville, USA), 3 g faecal sample collected from a *E. multilocularis* negative fox, 6 ml H&L (homogenization and lysis) buffer (100 mM Tris HCl pH 8.0, 5 mM EDTA pH 8.0, 0.2% SDS, 200 mM NaCl) and 2 ml of 5 M NaCl buffer.

### 2.2 Faecal samples from foxes naturally infected with *E. multilocularis* tested by Intestinal Scraping Technique (IST)

Faecal samples were taken from the ampulla recti/rectum of 120 naturally infected foxes collected in Brandenburg, Germany (Tackmann et al., 2001; Staubach et al., 2011). Based on the IST results, the samples were attributed to five groups (Table 1) (Maksimov et al., 2017).

**Table 1:** Faecal samples from foxes naturally infected with *Echinococcus multilocularis* tested by the Intestinal Scraping Technique (IST).

Sample groups	Number of samples	Worm burden according to IST	Additional information
−group	30	Negative	–
+group	30	1–5 worms	28 faeces from foxes with patent *E. multilocularis* infections and for two samples the status was not assigned
++group	30	>5–50 worms	28 faeces from foxes with patent *E. multilocularis* infections and two samples from foxes with prepatent infections
+++group	20	>50–1000 worms	19 faeces from foxes with patent *E. multilocularis* infections and one sample from a fox with prepatent infections
++++group	10	>1000 worms	all samples except one derived from animals with patent infections; for one fox, the status was not assigned

### 2.3 DNA extraction protocols

#### 2.3.1 Pre-extraction treatment of samples

Regardless of the applied DNA extraction or capture technique, 3 g of each faecal sample were initially used. To disrupt the cells or parasite eggs and to release the target DNA, each sample was mixed with 800 mg of 0.5 mm zirconium oxide beads, 600 mg of 2 mm zirconia beads (BioSpec Products Inc., Bartlesville OK, USA), 9 ml of homogenization and lysis buffer (100 mM Tris HCl pH 8.0, 5 mM EDTA pH 8.0, 0.2% SDS, 200 mM NaCl) and 3 ml of 5 M NaCl in a 15 ml Sarstedt screw cap tube. The sample was then bead-beaten using a FastPrep homogenizer (MP Bio, Santa Ana CA, USA) equipped with the TeenPrep 12 × 15 ml adapter. Shredding was carried out six times for 60 s, each time at maximum speed of 6.5 m/s. The tubes were then centrifuged for 10 min at 3000*g* without breaks (Centrifuge 5810 R, Eppendorf, Hamburg, Germany), the supernatants recovered and used immediately or after storage at −20 °C. Each run included a negative extraction control used throughout DNA extraction.

#### 2.3.2 Magnetic capture based DNA extraction protocol

A genome region in the mitochondrial DNA of *E. multilocularis* was targeted by magnetic capture (MC) (Isaksson et al., 2014). The supernatants (appr. 8 ml) were collected into fresh 15 ml Sarstedt screw cap tubes, where free biotin was removed. High performance streptavidin sepharose (GE Healthcare, Little Chalfont, UK) with a binding capacity of 300 nmol/ml was prepared and 100 μl added to each sample (Isaksson et al., 2014). The streptavidin sepharose beads were then separated by centrifugation of the samples at 3500*g* for 15 min without breaks (Centrifuge 5810 R, Eppendorf, Hamburg, Germany). To hybridize the biotinylated capture probe “EmFishF” (Table 2) to the target DNA, the supernatant was transferred into another fresh 15 ml tube, 15 μl (1 μM) biotinylated probe added and the sample incubated at 98 °C for 15 min in a water bath (VWR International GmbH, Hannover, Germany), followed by 45 min in a shaking (300 rpm) water bath (55 °C) (VWR International GmbH, Hannover, Germany). Finally, the samples were rotated (300 rpm) on a rocking platform for 15 min at room temperature. The probe/target DNA complexes were captured with paramagnetic beads and manually washed as described (Isaksson et al., 2014).

**Table 2:** Primers, probes, targets and sequences.

Primer/Probe (type of reagent)	Target	Protocol	Sequence	Reference
EmFishF (capture probe)	ND1	Magnetic capture	Biotin TEG-AAGAATTTTTATTTTCAAAGTCGTAGGTATATTGGTTTGTTGGGYGTTTTTTTGTTAATAATTTTGGTTATTATATATTCTTTTATTTATGGTAGATATTATAGTGTTAGTTA TAATAGT	Isaksson et al. (2014)
EmMGB_F (Forward primer)	12 s	MGBqPCR	GTGCTGCTYATAAGAGTTTTTG	Isaksson et al. (2014)
EmMGB_R (Reverse primer)	12 s	MGBqPCR	CTATTAAGTCCTAAACAATACCATA	Isaksson et al. (2014)
EmMGB_P (MGB probe)	12 s	MGBqPCR	VIC-ACAACAATATTCCTATCAATGT-MGB	Isaksson et al. (2014)
Em-rrn_F (Forward primer)	rrnL	TaqMan-qPCR	CTGTGATCTTGGTGTAGTAGTTGAGATTT	Knapp et al. (2014)
Em-rrn_R (Reverse primer)	rrnL	TaqMan-qPCR	GGCTTACGCCGGTCTTAACTC	Knapp et al. (2014)
Em_P (Probe)	rrnL	TaqMan-qPCR	FAM-TGGTCTGTTCGACCTTTTTAGCCTCCAT-TAMRA	Knapp et al. (2014)

The washed beads were resuspended in 100 μl TE-puffer and incubated for 10 min at 99 °C in a thermomixer at 300 rpm (Centrifuge 5810 R, Eppendorf, Germany) to release the captured *E. multilocularis* target DNA from the probe. To recover the target DNA, the beads were pelletized using the tube magnet (Qiagen, Hilden, Germany) and the TE-buffer containing target DNA transferred into a fresh 2 ml tube.

#### 2.3.3 DNA extraction using commercial kits

The NucleoMagVet kit (Macherey-Nagel, Düren, Germany) in combination with the King Fisher 96 Flex (Thermo Fisher Scientific, Braunschweig, Germany) (NM) was validated with 200 μl aliquots of supernatants obtained by bead-beating of 3 g faecal sample prepared as described in section ‘Pre-extraction treatment of samples’. Extraction was performed according to the kit manual (Table 2, protocols **B** and **D**).

In addition, the QIAamp Fast DNA Stool Mini Kit was also applied to 200 μl aliquots of the supernatants, which had been stored frozen after beat-beating. Extraction was performed as described in the QT-qPCR protocol as published (Maksimov et al., 2017) (Table 2, protocol **C**).

### 2.4 PCR amplification of the target DNA

DNA samples extracted using the MC protocol were tested for the presence of *E. multilocularis* genome fragments by MGBqPCR (Isaksson et al., 2014), where the probe was conjugated with the minor groove binder (MGB). MGB represent crescent-shaped molecules that selectively bind non-covalently to the minor groove of DNA, which allows higher melting temperatures and increases the specificity of the probe (Kutyavin et al., 2000). The protocol was carried out with a few modifications (Table 3, protocol **A**). All DNA samples were tested by adding 7.5 μl of QuantiTect® Multiplex PCR NoROX Kit (200 × 50 μl reactions, QIAGEN, Hilden, Germany), 400 nM of each of the primers EmMGB_F and EmMGB_R, and 133 nM of the hydrolysis probe EmMGB_P (Table 1, Table 2). Reactions were run in a total volume of 15 μl, of which 2 μl were template DNA retrieved from the DNA extraction. The MGBqPCR was carried out in a Bio-Rad CFX 96 Real-Time Detection System (Hercules, Bio-Rad Laboratories GmbH, Munich, Germany) using 50 °C for 2 min followed by an initial denaturation step at 95 °C for 10 min and 45 amplification cycles of 95 °C for 15 s followed by annealing and elongation at 60 °C for 1 min. Fluorescence was measured at the end of each cycle.

**Table 3:** Protocols for DNA extraction and their combinations with real time PCR techniques.

Protocol	Sample homogenization, preparation	DNA extraction	PCR	Reference
A	Bead-beating, centrifugation, immediate use	Magnetic capture from supernatant by specific probe EmFishF (Table 1)	MGBqPCR, real time PCR with MGB probe (Table 2)	Isaksson et al. (2014)
B	Bead-beating, centrifugation, frozen −20 °C until used	NucleoMagVet kit from supernatant	MGBqPCR, real time PCR with MGB probe (Table 2)	This study
C	Bead-beating, centrifugation, frozen −20 °C until used	QIAamp® Fast DNA Stool Mini Kit, QIAGEN from supernatant	TaqMan-qPCR (Knapp et al., 2014), using QuantiTect™	Maksimov et al. (2017)
D	Bead-beating, centrifugation, frozen −20 °C until used	NucleoMagVet kit from supernatant	TaqMan-qPCR (Knapp et al., 2014), using QuantiTect™	This study

Samples, from which DNA had been extracted by the NM method, were tested by MGBqPCR and TaqMan-qPCR as described (Maksimov et al., 2017) (Table 2, protocols **B** and **D**).

A TaqMan-qPCR amplifying a sequence from the large ribosomal RNA gene of *E. multilocularis* was used as an alternative protocol (Knapp et al., 2014; Maksimov et al., 2017) (Table 2, protocols **C** and **D**). In each 20 μl aliquot of the reaction volume, 10 μl qPCR master mix (QuantiTect), 500 nM rrnL primers (Em-rrn_F and Em-rrn_R), 160 nM hydrolysis probe (Em_P) and 5 μl DNA template were included (Table 1). The qPCR was carried out in a Bio-Rad CFX 96 Real-Time Detection System (Hercules, Bio-Rad Laboratories GmbH, Munich, Germany) using the cycling conditions previously described (Maksimov et al., 2017). Fluorescence was measured at the end of each cycle.

Each qPCR run included a negative extraction control used throughout DNA extraction, a negative PCR control (sterile deionized water), a positive control (*E. multilocularis*-DNA) and an internal control as previously described (Maksimov et al., 2017). All DNA samples were tested one time in the respective PCR protocol.

The evaluation of real time PCR results was performed using the Bio-Rad CFX Manager 3.0 software (Bio-Rad Laboratories GmbH, Munich, Germany).

The Cq value cutoffs in all PCRs were applied as described (Isaksson et al., 2014). Samples were considered negative if there was no Cq value or if there was a non-exponential growth of the qPCR curve (Caraguel et al., 2011).

### 2.5 Statistical analysis

For statistical computing and graphical presentation of the results, R (version 3.5.1) was applied (Team, 2018). For statistical comparison of Cq values between the protocols, the Mann-Whitney test from the R package “stats” was used.

To calculate 95% confidence intervals (CI) for diagnostic sensitivity, the function “*epi*.conf” from the R package “epiR” applied.

To test the null hypothesis (i.e. no difference) in any pair of proportions between several groups, the “pairwise.fisher.test” function from the package “fmsb” was applied. To adjust for type I error for multiple comparisons, a Bonferroni-correction was conducted (Bonferroni, 1936). In this test, a Bonferroni-corrected p-value of ≤0.05 was considered as statistically significant. Graphical presentation of the results was performed using the R package “ggplot2”.

## 3 Results

### 3.1 Analytical sensitivity

**Fig. 1:**
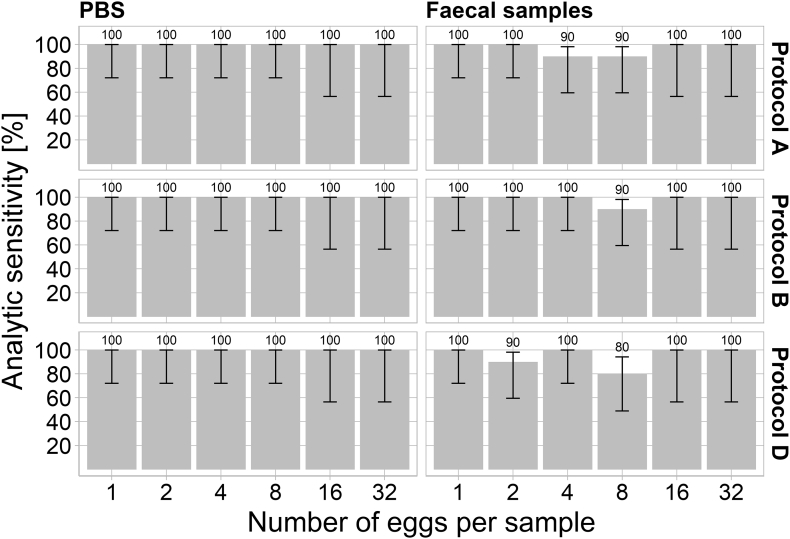
Analytic sensitivity of protocols A (Magnetic capture + MGBqPCR), B (NucleoMag Vet + MGBqPCR), and D (NucleoMag Vet + TaqMan-qPCR) stratified by the numbers of *Echinococcus multilocularis* eggs used for spiking. The estimated analytical sensitivities (grey bars and figures) and the respective 95% confidence intervals (whiskers) are shown.

Protocols A, B and D (Table 3) detected target DNA in all PBS samples (n = 50) spiked with various numbers of eggs (Fig. 1).

In the case of faecal samples spiked with defined numbers of eggs, the target DNA could be extracted and amplified by protocol A in most samples. Two samples spiked with four or eight eggs, respectively, were false-negative (Fig. 1).

Only one faecal sample spiked with eight eggs was false-negative by protocol B (Fig. 1). Protocol D failed to detect *E. multilocularis* target DNA in three faecal samples (Fig. 1). One of them was spiked with two eggs and another two samples with eight eggs (Fig. 1).

**Fig. 2:**
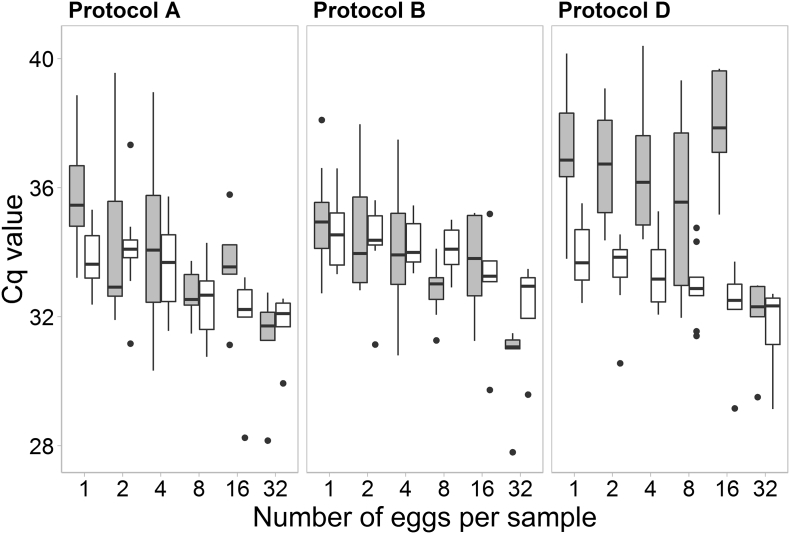
Cq values obtained in test runs performed for determining the analytical sensitivity of protocols A (Magnetic capture + MGBqPCR), protocol B (NucleoMag Vet + MGBqPCR) and D (NucleoMag Vet + TaqMan-qPCR). Grey boxes represent faecal samples and white boxes PBS samples, all spiked with defined numbers of *Echinococcus multilocularis* eggs. Black dots show outliers. The black bold line in each box represents the median. The boxes illustrate the 25–75% percentiles. The lower and upper whiskers stand for the range (minimum and maximum).

With respect to analytical sensitivity, the Cq values obtained with the experimentally spiked faecal samples in protocol D were markedly higher as compared to the Cq values from PBS samples processed in the same protocol, but also in comparison with the remaining Cq values obtained in protocols A and B (Fig. 2). However, none of the differences observed by pairwise comparison was statistically significant (Pairwise Mann-Whitney Test: Bonferroni corrected p-value > 0.05).

There were no significant differences between the protocols with respect to the numbers of samples classified as *E. multilocularis*-positive (Pairwise Fisher test: Bonferroni corrected p-value > 0.05).

### 3.2 Diagnostic sensitivity relative to IST

#### 3.2.1 Overall diagnostic sensitivity relative to IST

The highest overall diagnostic sensitivity (93% [95% CI: 86–97%]) relative to the IST was recorded for protocol A, followed by protocols C (89% [95% CI: 80–94%]) and B (86% [95% CI: 77–93%]). The protocol D showed the lowest overall diagnostic sensitivity (72% [95% CI: 62–82%]). The differences between the protocols were not statistically significant (Pairwise Fisher test: Bonferroni corrected p-value > 0.05).

#### 3.2.2 Diagnostic sensitivities and IST categories

**Fig. 3:**
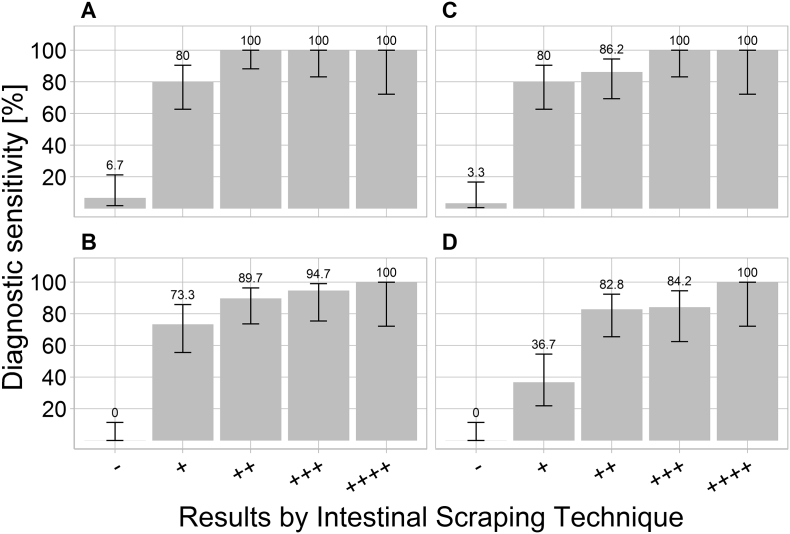
Diagnostic sensitivity of protocols A (Magnetic capture + MGBqPCR), B (NucleoMag Vet + MGBqPCR), C (QIAamp + TaqMan-qPCR) and D (NucleoMag Vet + TaqMan-qPCR) versus Intestinal Scraping Technique (IST). The validation was performed with 120 faecal samples derived from foxes with different *Echinococcus multilocularis* worm burdens as determined by IST. The worm burdens were assigned to 5 different categories (−, +, ++, +++, ++++; see Material and methods). The estimated analytical sensitivities (grey bars and figures) and the respective 95% confidence intervals (whiskers) are shown.

The diagnostic sensitivity (proportion of positive results obtained by the respective protocol relative to the IST results) between the IST categories varied between 36.7% and 100% (Fig. 3).

Protocol A detected *E. multilocularis* DNA in all samples of the IST categories “++”, “+++” and “++++”. In samples of the IST category “+”, protocol A detected *E. multilocularis* DNA in 80.0% of the samples. In the group of *E. multilocularis*-negative samples, protocol A was positive in 6.7% of the samples (Fig. 3).

Protocol C yielded the second-highest diagnostic sensitivity; *E. multilocularis* DNA was amplified in all samples of the categories “+++” and “++++”. In the groups “+” and “++”, 80.0% or 86.2% of the samples, respectively, were positive for *E. multilocularis* DNA. Among the IST negative samples, 3.3% samples were *E. multilocularis*-positive by this method (Fig. 3).

Protocols B and C detected *E. multilocularis* DNA only in group “++++” in all samples. The diagnostic sensitivity in the remaining IST *E. multilocularis*-positive categories varied between 46.7% (“+” with protocol C) and 94.7% (“+++” with protocol B) (Fig. 3).

No statistically significant differences in diagnostic sensitivity were found for the protocols when stratified by IST category (Pairwise Fisher test: Bonferroni corrected p-value > 0.05).

**Fig. 4:**
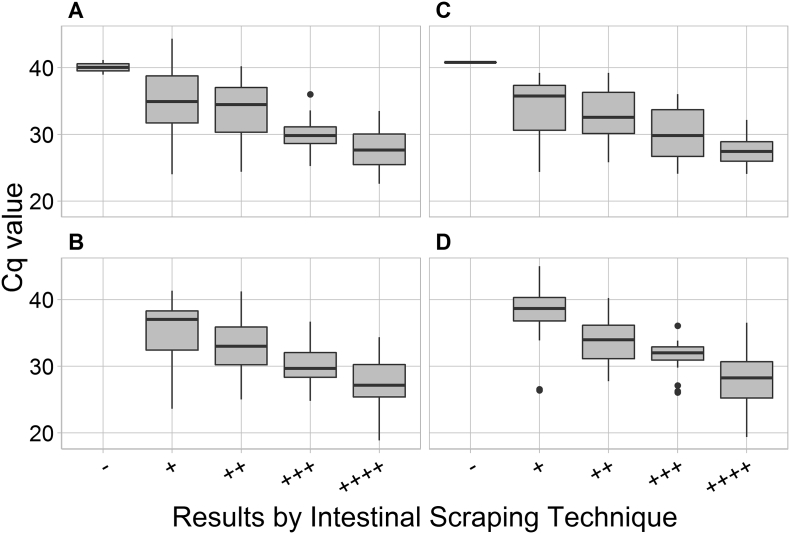
Cq values obtained by protocols A (Magnetic capture + MGBqPCR), B (NucleoMag Vet + MGBqPCR), C (QIAamp + TaqMan-qPCR) and D (NucleoMag Vet + TaqMan-qPCR) for 120 faecal samples derived from foxes. The samples had been examined by the Intestinal Scraping Technique (IST) before DNA was extracted and were assigned to different categories of *Echinococcus multilocularis* worm burdens (−, +, ++, +++, ++++; see Material and methods). Black dots show outliers. The black bold line in each box represents the median. The boxes illustrate the 25–75% percentiles. The lower and upper whiskers stand for the range (minimum and maximum).

Moreover, no statistically significant differences in the Cq values between the methods were recorded (Fig. 4).

## 4 Discussion

The findings of the present study demonstrate that the magnetic DNA capture protocol published by Isaksson et al. (2014) (protocol A) can also be used as an alternative method for the diagnosis of *E. multilocularis* in the final host, instead of or in addition to the Intestinal Scraping Technique. We have also shown that protocol C, which is based on a commercial extraction kit in combination with a TaqMan real time PCR and on processing the supernatants of homogenized samples, yielded a similar diagnostic sensitivity as the magnetic DNA capture protocol and may represent a less expensive and faster alternative as compared to protocol A.

The IST and the SCT represent frequently used standard methods for the diagnosis of *E. multilocularis* infections in final hosts of the parasite (Tackmann et al., 2006; Conraths and Deplazes, 2015). Yet, these methods are laborious and require parasitologically experienced staff. Moreover, they are only applicable post mortem and can therefore not be used for intra vitam diagnosis for example in pet dogs or cats. However, a number of techniques suitable for the intra vitam diagnosis of *E. multilocularis* infections were developed as reviewed by Conraths and Deplazes (2015).

PCR methods based on the amplification of DNA extracted from faecal samples may be suitable for the diagnosis of *E. multilocularis* in the final host. However, one of the disadvantages is the influence of co-extracted PCR inhibitors if faecal samples are used (Opel et al., 2010). Such inhibitors may lead to false- negative results, even in samples containing a large number of parasite eggs (Maksimov et al., 2017). Another limitation of PCR-based methods is the small amount of sample (0.5 g) that can be processed in the available commercial DNA extraction kits, which can also lead to false-negative results, at least partially also due to an unequal distribution of parasite eggs in the sample. Isaksson et al. (2014) developed an MC-based DNA extraction method (protocol A), which is much less prone to inhibition of the amplification of the target DNA and can be used to process about 3 g of faecal matter. Oines et al. (2014) showed that analytical sensitivity of this method is higher, especially in faecal samples spiked with small numbers of eggs, if compared to a flotation and sieving method.

The MC method was originally validated with vulpine faecal samples that were also tested by SCT. The performance of this protocol relative to IST is not known. In the present study, the MC protocol was validated with samples that had been characterized by an IST version, which has also been validated against the SCT (Tackmann et al., 2006). We also tested commercial DNA extraction protocols with aliquots from bead-beaten samples: i. NucleoMag Vet kit combined with two different real time PCRs (protocol B and D) as an alternative to the MC-MGqPCR method; and ii. a combination of QIAamp DNA extraction kit with a TaqMan-qPCR (protocol C), which had been validated (termed QT-qPCR protocol) in a previous study (Maksimov et al., 2017).

The MC-qPCR is markedly less laborious as compared to the classical parasitological methods IST and SCT. About 30 faecal samples can be processed within two days, even if manual washing steps are performed. The sample throughput of the MC-qPCR depends on the available equipment, i.e. in our laboratory the FastPrep homogenizer with the respective adapter, two water baths that heat samples to a temperature of 99 °C (Isaksson et al., 2014). The required reagents are relatively expensive (appr. 10.62 € per sample including the costs for Zirkonium beads, EmCaptBiotProbe, Dynabeads and Streptavidin Sepharose). While the NucleoMag Vet protocols (B and C) allow for a much higher sample throughput (appr. 30 min for DNA extraction of up to 96 samples) if a fully automated DNA extraction apparatus is available. This extraction protocol is also based on paramagnetic beads (non-specific DNA binding), which, according to the suppliers, should contribute to a reduced co-extraction of copro-inhibitors. The method is much cheaper (appr. 2.7 € extraction costs per sample) relative to the MC DNA extraction protocol (A).

Combinations of DNA extraction and PCR amplification protocols were first tested for their analytical sensitivity. The results showed that MC (protocol A), but also the NucleoMag Vet protocols (B and D) perform well and detect *E. multilocularis* DNA in spiked PBS samples, but also in most of faecal samples spiked with varying defined numbers of eggs. The QT-qPCR protocol (protocol C) had already been validated in a previous study (Maksimov et al., 2017). The analytical sensitivity results obtained for the MC method (protocol A) were similar to that found by Isaksson et al. (2014).

No significant differences regarding the analytical sensitivity of the examined DNA extraction methods were observed. Possible explanations for false-negative results in spiked faecal samples may include the negative influence of co-extracted copro-inhibitors (Opel et al., 2010; Maksimov et al., 2017) on the amplification process or the loss of target DNA due to its potential adhesion to the wall of tubes (Gaillard and Strauss, 1998).

Our results also show that the MC method (protocol A) had the highest diagnostic sensitivity (93%), but the differences between the tested protocols was not statistically significant. Further studies with higher sample size are needed to confirm these results. A similar diagnostic sensitivity (88%) was also reported for the MC protocol relative to SCT (Isaksson et al., 2014).

Probably due to co-extraction of inhibitors, false-negative results were obtained in all protocols involving commercial DNA extraction kits. Most of the false-negative DNA samples obtained with these protocols had a rusty color, which may indicated the presence of heme, a known PCR inhibitor. In some cases, diluting such samples 1:10 and 1:100 led to the amplification of *E. multilocularis* DNA (data not shown). Surprisingly, the lowest diagnostic sensitivity was obtained with the NucleoMag Vet kit (protocol D), which was probably also due to the influence of co-extracted inhibitors. This DNA extraction kit was apparently not able to remove PCR inhibitors in all DNA samples, at least not in our hands. Including additional PCR inhibitor clean up kits may improve the performance of this DNA extraction kit, but also of the kit used in protocol C. However, this needs to be tested in further experiments. It is noteworthy that the amount of material processed differed between the DNA extraction kits. In the MC-qPCR protocol, where the whole supernatant of the sample was used for extracting the target DNA, only 200 μl of this supernatant can be analyzed with the applied kits. This difference may have also reduced the sensitivity in the detection of target DNA.

In a previous study, a QT-qPCR protocol (protocol C in the present study) revealed a diagnostic sensitivity of 81%, when DNA was directly extracted from 0.2 g of each faecal sample (Maksimov et al., 2017). In the present study, a diagnostic sensitivity of 89% was recorded for this protocol, when 3 g from the same faecal samples were lysed and bead-beaten, and an aliquot was further proceeded for DNA extraction. These results suggest that using aliquots from 3 g of bead-beaten faecal samples for PCR amplification (Isaksson et al., 2014) may increase the diagnostic sensitivity of commercial DNA extraction protocols, at least in the case of the QIAamp DNA extraction kit.

The applied real time qPCR protocol also seems to play a significant role in the detection of the target DNA. Our results show that the NucleoMag Vet extraction protocol together with the MGB.qPCR (protocol B) (Isaksson et al., 2014) had a higher diagnostic sensitivity as compared to the combination of the same extraction technique with a TaqMan-qPCR (protocol D). The PCR amplification kit can be excluded as the cause for this difference, as the same PCR amplification kit was used in both PCR protocols (i.e. MGBqPCR and TaqMan-qPCR).

The MC and QIAamp-based protocols A and C detected *E. multilocularis* DNA in 6.7% or 3.3% of the *E. multilocularis* negative group of samples, respectively. Since these samples had been characterized by IST, which has diagnostic sensitivity of appr. 80% (Conraths and Deplazes, 2015), we assume that the samples may have been false-negative in the IST, perhaps due to a very low worm burden, or the *E. multilocularis* DNA detected in these samples may have originated from digested metacestodes. Finally, false-positive PCR results cannot be completely ruled out, but seem less likely. These results should be also taken into account when faecal samples of pets are analyzed for the presence of *E. multilocularis* eggs.

Some limitations of the present study should also be mentioned here. The values for the diagnostic sensitivity of the tested protocols refer only to patent *E. multilocularis* infections in foxes. The diagnostic performance with regard to prepatent infections may differ and still needs to be established.

Since it was not possible to obtain as sufficient number of well characterized faecal samples from *E. multilocularis*-infected dogs for validation purposes, only faecal samples of foxes were used, which represent the main final host for *E. multilocularis* in Central Europe. However, we believe that the performance of the protocols may be comparable if canine faecal samples are used.

Although our study demonstrates that the protocols based on the detection of *E. multilocularis* DNA in faecal samples of foxes are promising, these methods cannot replace conventional parasitological methods completely. The dependence of the DNA detection protocols on components provided by various commercial suppliers represents a limitation of these methods. When components are modified or taken from the market, the respective diagnostic protocols will have to be revalidated or replaced by other techniques. It should also be noted that the DNA capture methods are relatively expensive and may therefore not be applied in some laboratories for diagnostic or monitoring purposes.

Upon appropriate adaption and validation, the protocols described in our study may also be applicable for the diagnosis and monitoring of other members of the genus Echinococcus, e.g. the *E. granulosus* sensu lato complex in dogs.

## 5 Conclusion

The results of our study suggest that MC-based protocols may be suitable for diagnosing *E. multilocularis* infections in foxes and for monitoring the epidemiological situation regarding this infection in its main final host. Probably the method can also be used to examine other definitive hosts such as pet dogs and cats. The QIAamp DNA extraction kit together with TaqMan-qPCR had a similar diagnostic sensitivity as the DNA capture–based protocols, when aliquots of 3 g of bead-beaten faecal samples were analyzed. None of the commercial DNA extraction kits was able to remove PCR inhibitors sufficiently from all samples, so that false-negative results were obtained with the respective samples. It may be useful to include in the commercial DNA extraction protocols additional steps to remove or inactivate PCR inhibitors.

## Conflict of interests

The authors report no conflict of interest.
